# Workload demands, surgical education and training among resident surgeons in Germany: a cross-sectional questionnaire-based study

**DOI:** 10.1186/s12909-026-10519-w

**Published:** 2026-10-19

**Authors:** Hans C Schmidt, Melanie Le, Michael Sennert, Kira Meißner, Laetitia Hampe, Anas A Preukschas, Felix Nickel, Mara Goetz, Philipp A Wise, Amila Cizmic

**Affiliations:** 1 SimLab – Students for Surgery, University Medical Center Hamburg- Eppendorf, Hamburg, Germany; 2 Department of Pediatric Surgery, University Medical Center Hamburg- Eppendorf, Hamburg, Germany; 3 Department of Pediatric Surgery, Sana Clinic Offenbach, Offenbach, Germany; 4 Department of General, Visceral and Thoracic Surgery, University Medical Center Hamburg-Eppendorf, Martinistraße 52, Hamburg, 20251 Germany; 5 Department of Neuroradiology, University Hospital Heidelberg, Heidelberg, Germany

**Keywords:** Workload in surgery, Resident surgeons, University hospitals, Surgical training

## Abstract

**Background:**

The medical system is experiencing increasing systemic workload pressures and a developing shortage of doctors, making the well-being of resident surgeons a critical factor for sustainable healthcare delivery. By highlighting the surgical residents’ perspectives through a direct questionnaire, the current study aimed to provide an exploratory basis for discussing reform possibilities that better support current and future demands on surgeons.

**Methods:**

This cross-sectional study used a custom-developed questionnaire to collect data from resident surgeons across multiple surgical specialties, predominantly at university hospitals in Germany. It was designed to assess the quality of resident surgeons’ training experiences and gauge their perceptions of their career progression and workload distribution. Furthermore, the questionnaire assessed emotional well-being, workload demands, training sufficiency, satisfaction with career progression, and the allocation of time between administrative and patient care tasks. Data collection was conducted through an anonymous online questionnaire platform.

**Results:**

One hundred eighty-six surgical residents responded fully to the survey. Most participants worked excessive hours, with 44.1% working over 55 h weekly, and administrative tasks dominating their time (72.0% ranked first), compared to surgical procedures (4.3% ranked first). Structured training programs were lacking in 88.7%, and 74.2% reported insufficient surgical exposure. Professional readiness was mixed: 67.2% felt confident in their ability to adapt to emerging technology, but 51.0% felt unprepared for independent research. The career outlook appeared concerning, with 42.9% considering leaving within three years, and 67.7% pessimistic about work-life balance. The residents’ assessment of the healthcare system’s readiness for future challenges was poor, with 81.1% disagreeing that the healthcare system is prepared for surgical innovation and 88.7% citing insufficient staffing to meet demographic changes.

**Conclusion:**

The current view of surgical residency at university hospitals in Germany reveals gaps in structured training programs and surgical exposure, which could lead to staff shortages and insufficient preparation for independent research and professional fulfillment in the future.

**Supplementary Information:**

The online version contains supplementary material available at https://doi.org/10.1186/s12909-026-10519-w.

## Introduction

Job stress and burnout are significant concerns in the medical profession, particularly among resident surgeons who face intense work demands [1–3]. The well-being and job satisfaction of resident surgeons are directly linked to patient satisfaction, care, and the overall effectiveness of the healthcare system [4, 5].

As Germany’s population ages and a significant number of doctors approach retirement, the workload is expected to increase in all medical fields [6, 7]. Due to increased bureaucratic tasks and documentation, resident surgeons spend less time performing sub-steps of surgical procedures and entire surgeries independently in the operating room [8, 9].

Currently, 36 university hospitals and 1.874 hospitals with a total of 40.766 surgeons are involved in surgical healthcare in Germany (https://www.uniklinika.de/die-deutschenuniversitaetsklinika/), (https://de.statista.com/statistik/daten/studie/2617/umfrage/anzahl-der-krankenhaeuser-in-deutschland-seit-2000/). In addition to the existing imbalance between direct and indirect patient contact, such as administrative contact, high workloads are factors to be taken seriously to ensure a high level of job satisfaction, which has been proven to be promising for high-quality patient care [4, 8].

Reducing the factors that lead to burnout and specifically promoting potentially compensatory elements, such as academic development opportunities and structured training curricula, could help alleviate the burden on young surgeons [10]. Additionally, mentoring appears to be an effective tool for providing psychosocial support and facilitating identity formation in the clinical context [11]. Financial or academic incentives could also provide at least partial compensation for the high workload [12].

To address the issues mentioned, this study aims to explore the perspectives of resident surgeons at German university hospitals on their career progression and workload. Additionally, it seeks to describe the quality and structure of surgical training from the perspective of resident surgeons to gain insight into its current state.

The study aimed to assess the prevailing conditions of workload demands, surgical training and education, and career progression among resident surgeons affiliated with university hospitals in Germany.

## Materials and methods

The study was designed as a cross-sectional questionnaire-based study to gather data from resident surgeons across multiple surgical specialties in German university hospitals, and the survey was distributed to potential participants via email. It was approved by the local ethics committee (2024-300507-WF).

To ensure methodological rigor and adherence to recognized research practices for questionnaire-based studies, the internationally recognized research guideline, the Checklist for Reporting Results of Internet E-Surveys (CHERRIES), was followed (Supplementary Material 1) [13].

Before participating in the study, participants were required to provide informed consent, as indicated by the e-consent form available in the repository.

### Questionnaire

A custom-developed questionnaire aimed to assess the following dimensions of the resident surgeon’s job: emotional well-being, workload demands, training sufficiency, career progression satisfaction, and allocation of time between administrative and patient care. The distributed questionnaire can be found in Supplementary Material 2.

The questionnaire consisted of 30 questions and was distributed via an anonymous online survey platform (LimeSurvey). All questions provided a non-response option, and only one response option was selectable for each question. There was a comment section after every question, allowing residents to share their personal experiences and remarks.

The 30 questions covered the following topics:

1. Work-Life Balance: The questions evaluated how effectively resident surgeons balance their work and personal lives [14].
2. Resident Training Assessment: Questions aimed to quantitatively evaluate surgical training structure, including resident experiences, adequacy of hands-on practice, feedback quality, access to educational resources (training centers, labs, financial support for courses), and supervisor allocation and support.
3. Workload Allocation: This section of the questionnaire sought to understand how resident surgeons divide their time between patient care, administrative tasks, and other duties.
4. Resident Opinion on Career Progression and Future Workload: A series of questions was included to evaluate the resident surgeons’ perceptions of their career development and anticipated future workload.

### Study population

Resident surgeons at university hospitals in Germany were invited via mail to participate in the study voluntarily. The surgical specialties included were General and Visceral Surgery, Orthopaedic and Trauma Surgery, Vascular Surgery, Cardiothoracic Surgery, Neurosurgery, Maxillofacial Surgery, Plastic Surgery, Urology, Paediatric Surgery, and Gynaecology. Although the study’s emphasis was on assessing resident surgeons, attending, fellow, and senior surgeons could also participate in the survey, providing their assessments for comparison.

### Data collection

Data was collected through a web-based repository, LimeSurvey, ensuring anonymity and confidentiality of participants. The participants did not provide their personal data (names and date of birth) in the survey. The open survey questionnaires were accessed via a link or a QR code. The set time frame for data collection was between August 2024 and May 2025.

### Statistical analysis

Data analysis was performed using Python 3.7 with the following libraries: pandas for data manipulation, NumPy for numerical computations, Matplotlib and Seaborn for data visualization. Survey responses were analyzed using descriptive statistics, with categorical variables presented as frequencies and percentages. Descriptive statistics were calculated for all variables, including response counts, percentages, and 95% confidence intervals where appropriate.

Statistical significance testing was not performed as this was a descriptive cross-sectional survey focused on characterizing current conditions rather than hypothesis testing. Results are presented with corresponding sample sizes to facilitate interpretation and comparison with future studies.

## Results

A total of 285 responses were collected. However, 96 responses were excluded from the analysis for incompleteness, and 3 were excluded due to withdrawal from the data processing agreement. Additionally, due to the low response rate from the non-resident groups, the statistical analysis of these respondents was omitted.

### Demographics

**Fig. 1:**
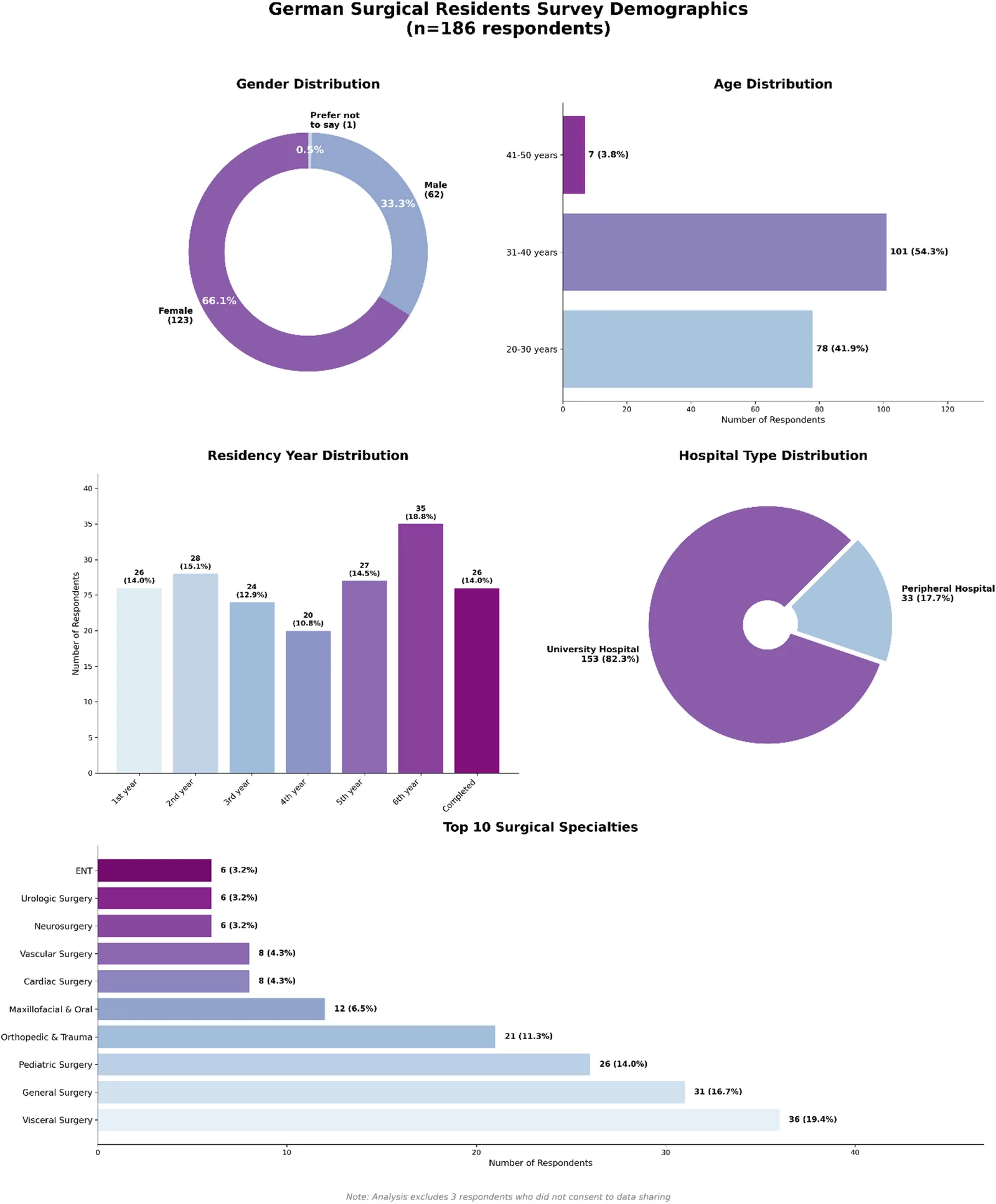
Demographic characteristics of German surgical resident survey respondents

The study included 186 surgical residents. The age distribution showed that 78 respondents (41.9%) were aged 20–30 years, 101 (54.3%) were aged 31–40 years, and 7 (3.8%) were aged 41–50 years. The gender distribution was 123 females (66.1%), 62 males (33.3%), and one who preferred not to say (0.5%). Most participants worked in university hospitals (153, 82.3%) versus peripheral hospitals (33, 17.7%). Residency status distribution included, in descending amount, sixth-year residents (35, 18.8%), second-year residents (28, 15.1%), fifth-year residents (27, 14.5%), residents with completed training (26, 14.0%), first-year residents (26, 14.0%), third-year residents (24, 12.9%), and fourth-year residents (20, 10.8%) (Fig. 1).

### Work time allocation, work load, and financial compensation

**Fig. 2:**
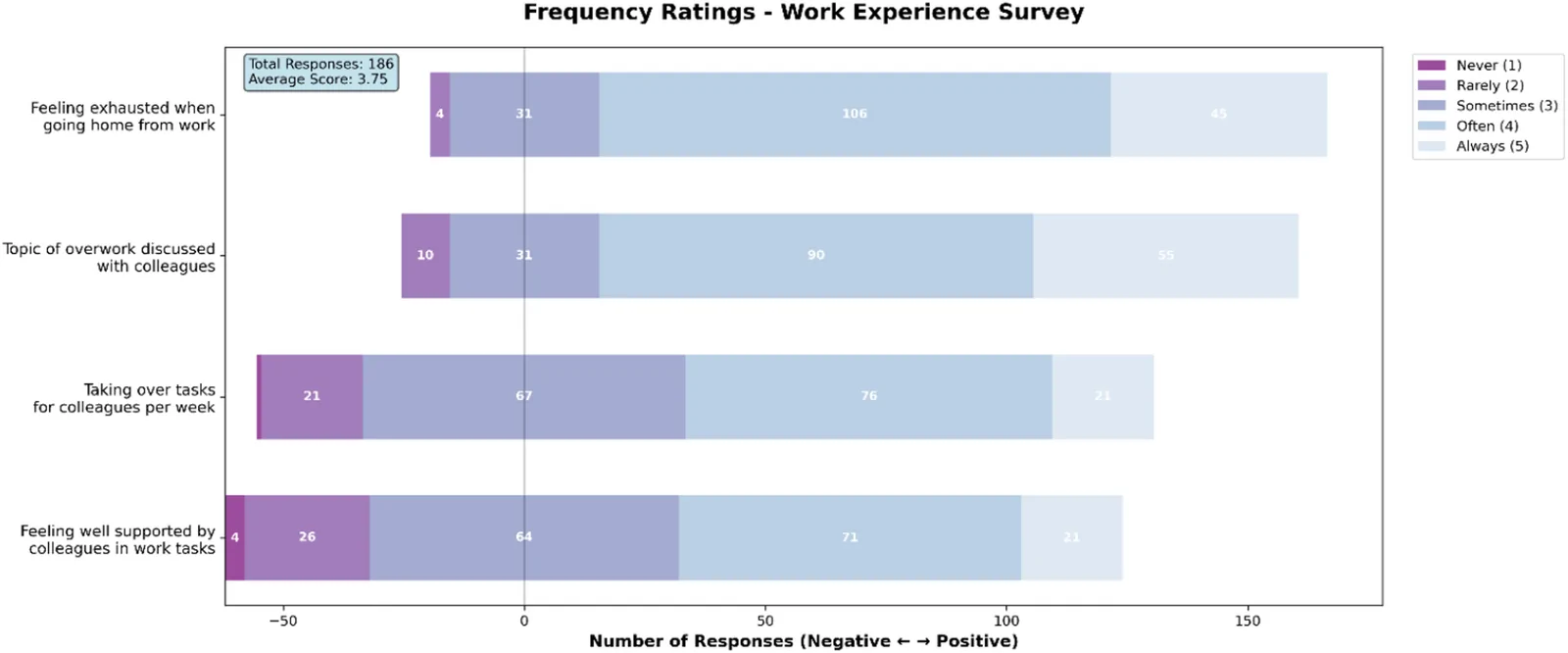
Quality of work experience ratings among surgical residents

The large majority of respondents reported working long hours, with 44.1% working an average of more than 55 h per week on a regular basis (Supplementary Material 3). Exhaustion upon returning home from work occurred frequently for 106 respondents (57.0%) and always for 45 respondents (24.2%). Colleague support showed that 26 respondents (14.0%) rarely felt supported and 64 respondents (34.4%) sometimes felt supported (Fig. 2).

Financial compensation adequacy was demonstrated by 47 respondents (25.3%) who strongly disagreed and 69 respondents (37.1%) who disagreed. The paid time off compensation showed that 72 respondents (38.7%) strongly disagreed, and 71 respondents (38.2%) disagreed (Supplementary Material 4).

**Fig. 3:**
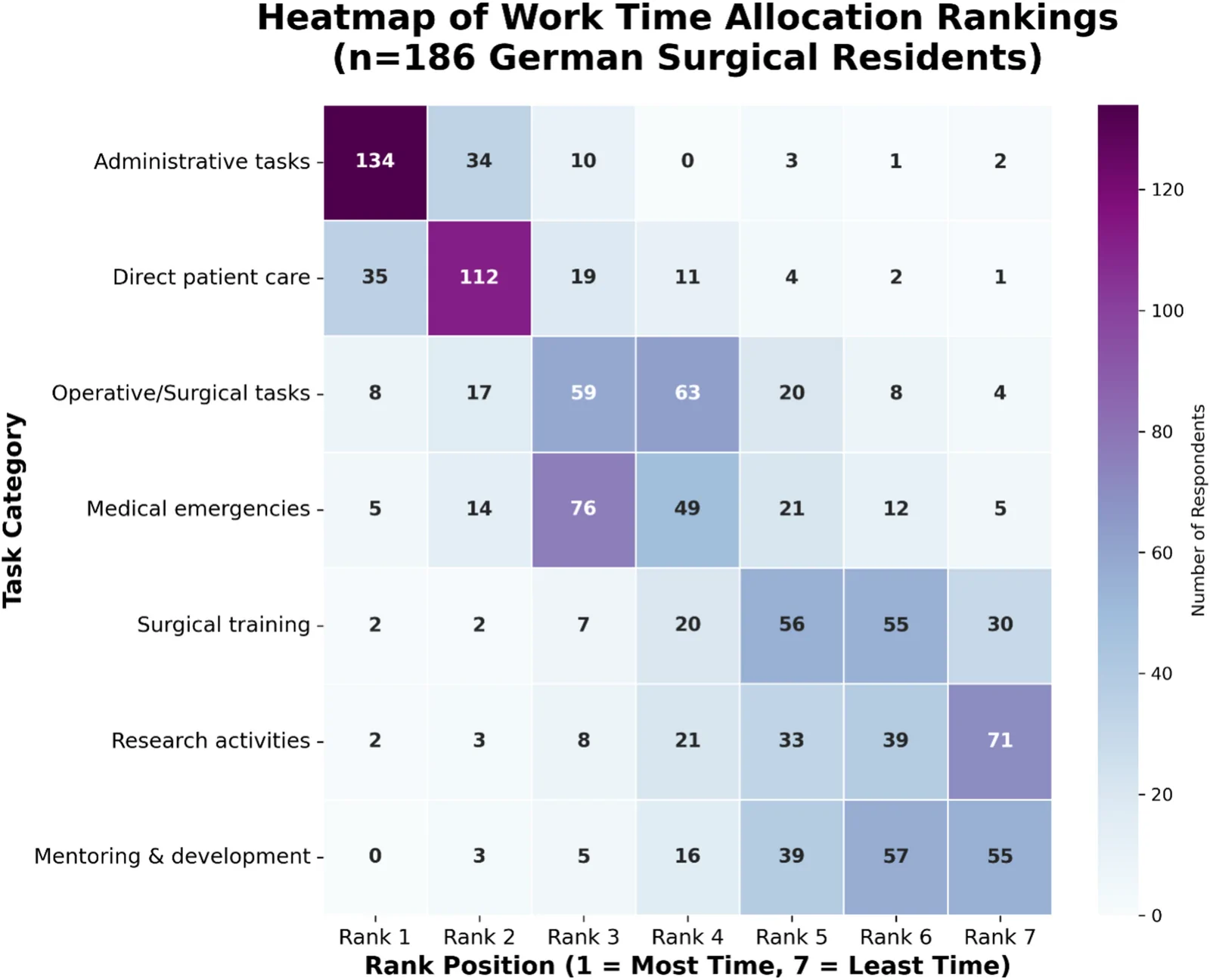
Work time allocation rankings by respondents

In a more granular analysis, work time allocation rankings revealed that administrative tasks and documentation were the top time requirements for 134 respondents (72.0%), while surgical procedures ranked first for only eight respondents (4.3%). Direct patient-related non-surgical tasks ranked second in time requirement (60.5%) while medical emergencies ranked third (41.3%). Surgical training ranked fifth time requirement for 56 respondents (31.8%) and sixth time requirement for 55 respondents (31.6%). Research activities ranked lowest (seventh rank) for 71 respondents (42.3%) (Fig. 3).

### Structured surgical training

**Fig. 4:**
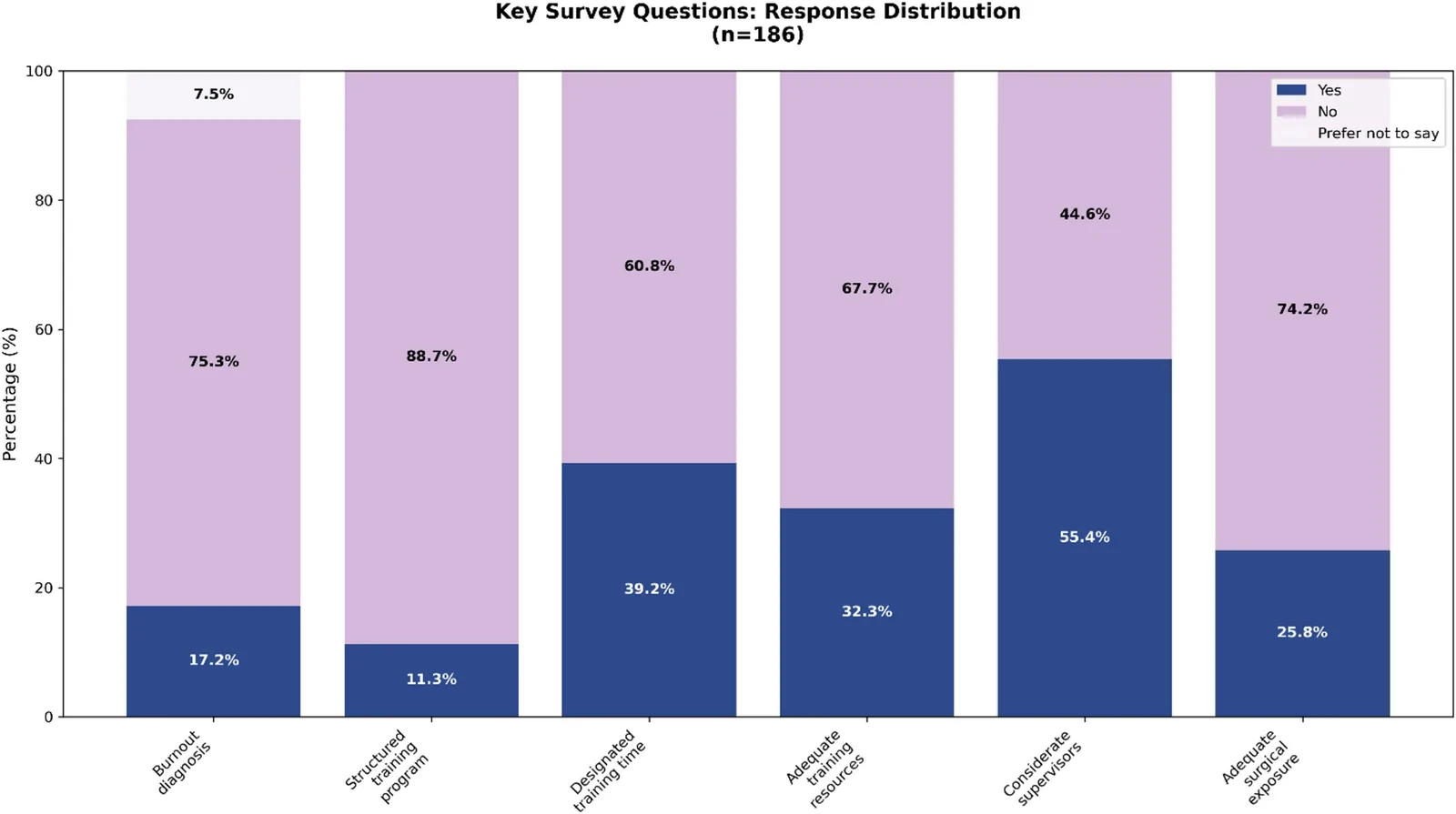
Response distribution for key survey questions of burnout and structured training program assessment

Structured surgical training programs were lacking for 88.7% of respondents. The designated time for access to the surgical training center was unavailable to 60.8% of respondents, and 30.0% reported that their hospital lacked a dedicated surgical training center. Surgical exposure was inadequate for 74.2% of respondents, while only 25.8% reported adequate exposure. 17.2% of respondents reported seeking professional help for a work-related health condition, such as burnout (Fig. 4).

**Fig. 5:**
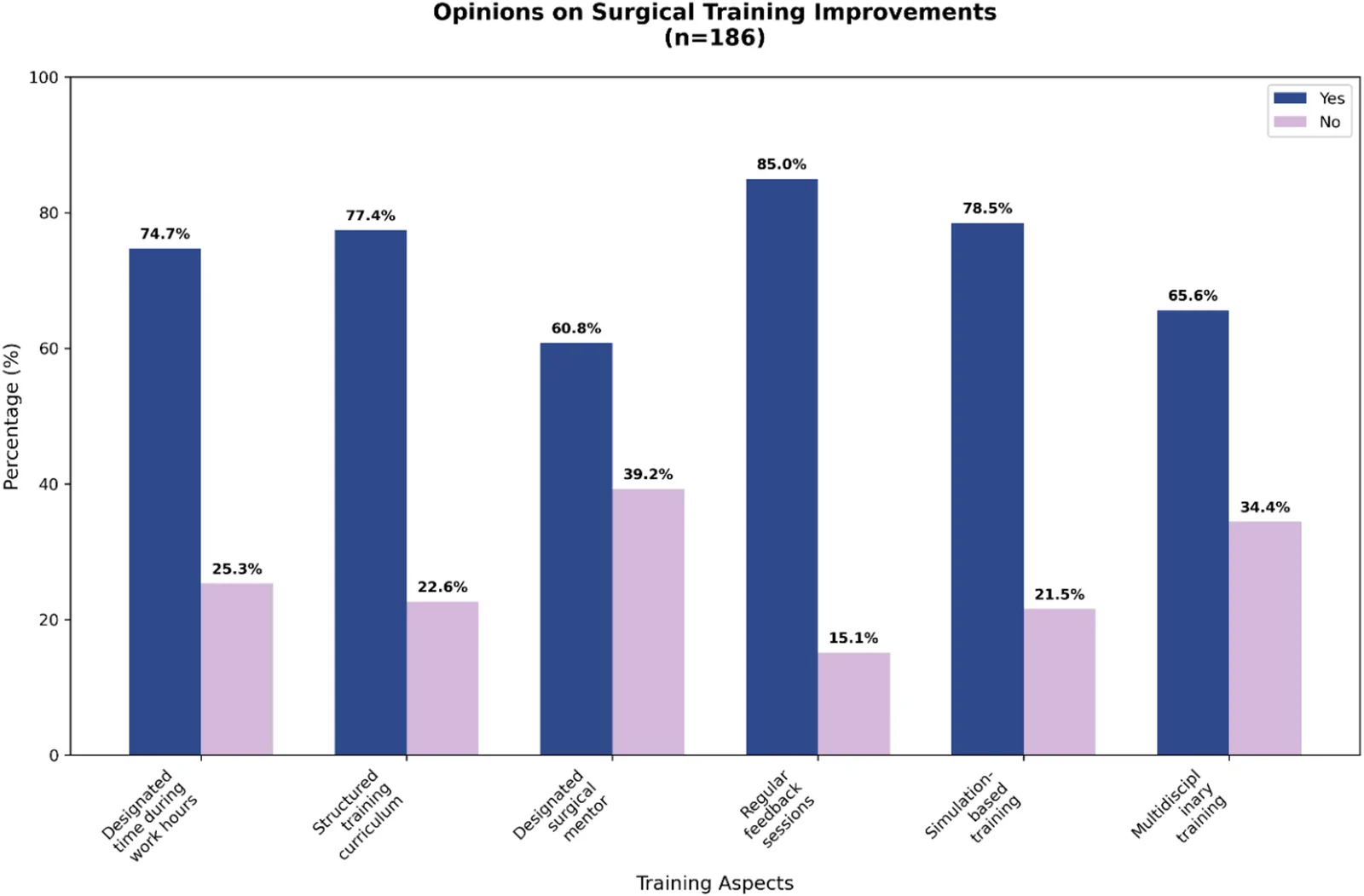
Opinions on surgical training improvements among respondents

Residents generally regarded the suggestions put forth in the survey on training curricula as favorable. The most requested suggestion was structured regular feedback during surgical training (Fig. 5).

### Professional readiness and ability assessment

**Fig. 6:**
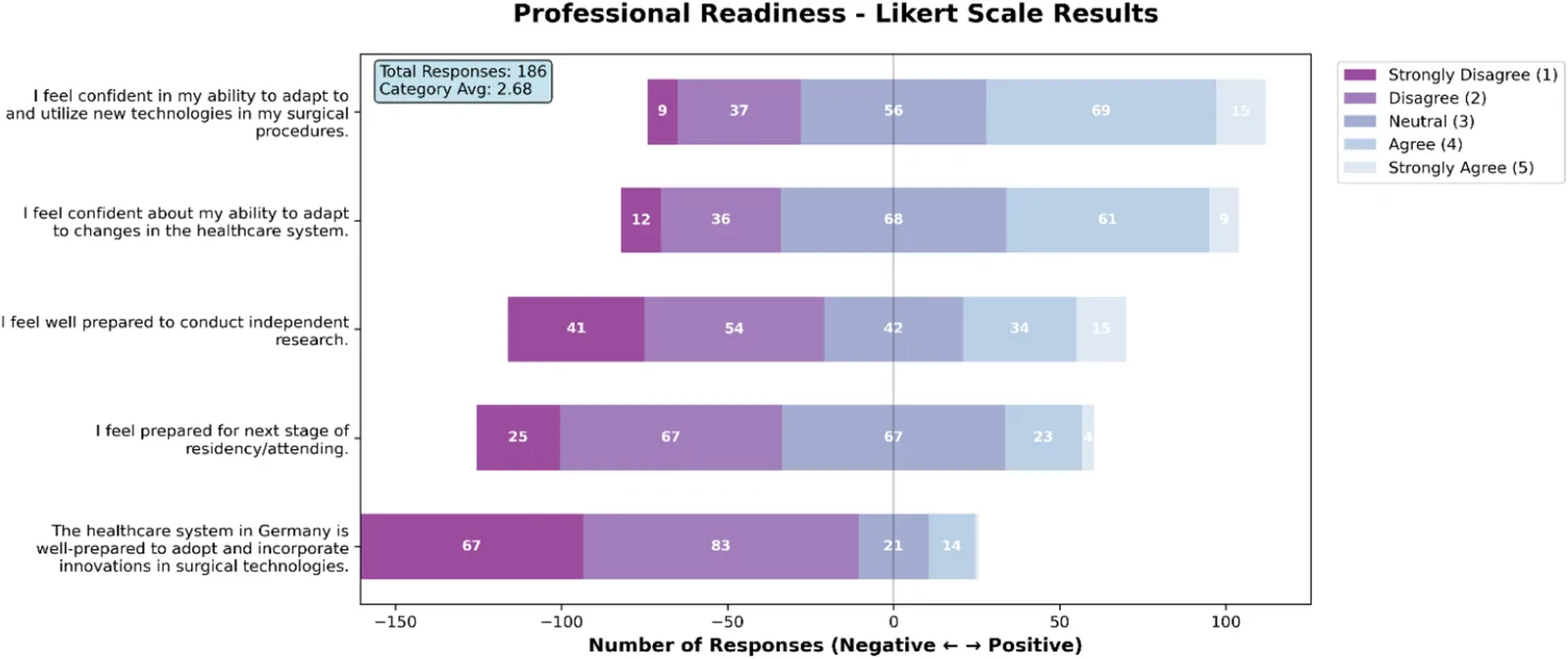
Professional readiness and ability assessment

Professional readiness evaluations showed mixed confidence levels across multiple domains. The technology adaptation confidence demonstrated a 67.2% agreement, indicating strong agreement. The healthcare system adaptation showed a 37.6% agreement. Research preparedness showed 51.0% disagreement or strong disagreement, while only 26.4% expressed confidence. Preparation for the next career stage showed 49.3% disagreement, with only 14.6% feeling prepared. Healthcare system preparedness for surgical technology innovation demonstrated 81.1% disagreement or strong disagreement (Fig. 6).

### Technology and workload impact

Technology integration showed varied impacts on resident workload. The digitization of patient files demonstrated a mixed response pattern, with 12 respondents (6.5%) rating the impact as very negative, 37 (19.9%) as negative, 67 (36.0%) as neutral, 52 (28.0%) as positive, and 18 (9.7%) as very positive. High-end surgical tools such as Da Vinci systems demonstrated a similar neutral predominance, with 13 respondents (7.0%) rating the impact as very negative, 23 (12.4%) as negative, 107 (57.8%) as neutral, 29 (15.7%) as positive, and 13 (7.0%) as very positive (Supplementary Material 5).

### Healthcare system and workforce outlook

Healthcare system and workforce outlook assessments revealed concerns about future capacity and staffing. The expected workload increases for surgeons showed 67.7% agreement, indicating strong agreement. The influence of telemedicine on surgical patient care demonstrated 69.0% agreement, indicating strong agreement. Staffing adequacy for technological changes showed 85.5% disagreement or strong disagreement. Staffing adequacy for demographic changes indicated 88.7% disagreement or strong disagreement (Supplementary Material 6).

### Career outlook and professional development

**Fig. 7:**
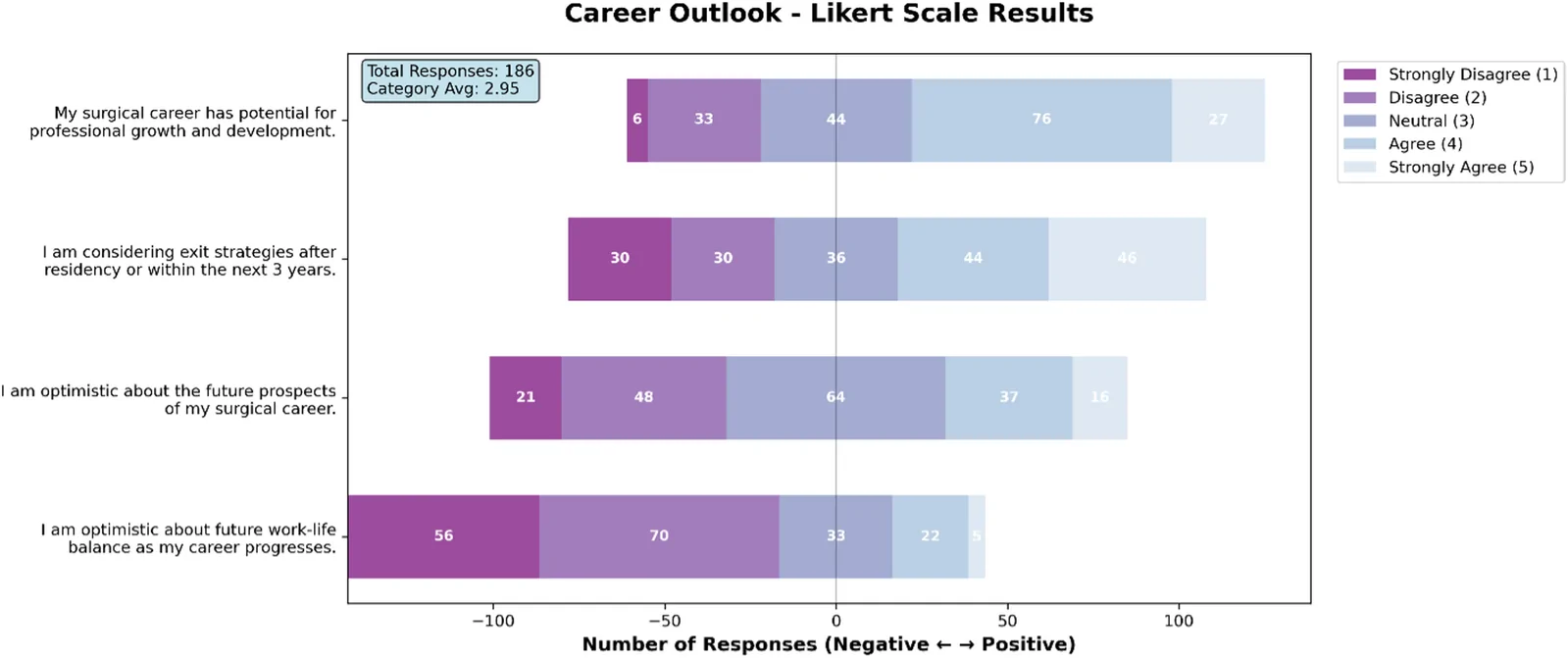
Career outlook assessment

Career outlook evaluations showed mixed perspectives on professional development and retention. Professional growth potential demonstrated 64.5% agreement or strong agreement. Exit strategy considerations within three years showed 42.9% agreement or strong agreement. Career optimism was reported by 37.1% of respondents as disagreement or strong disagreement, with 34.4% reporting neutral responses. Work-life balance optimism demonstrated 67.7% disagreement or strong disagreement (Fig. 7).

## Discussion

The presented cross-sectional questionnaire-based study provided an overview of surgical work-related conditions among resident surgeons at university hospitals in Germany. The findings demonstrated widespread dissatisfaction with current training structures, including limited access to structured surgical training programs and inadequate surgical exposure.

Workload concerns are prevalent, with administrative tasks accounting for a significant portion of time allocation. This correlates with the reporting from the USA, where Ellis et al. described similar results in surgical residents in the USA when reporting workload problems during surgical residency [15].

Professional readiness assessments reveal mixed confidence levels, particularly concerning research preparedness and preparation for career advancement. Most surgical residents believe there is insufficient staffing to manage technological changes and cite inadequate staffing for demographic transitions in an aging population. High workload, long working hours, and personal mistreatment are key factors associated with burnout and suicidal thoughts in surgical resident training [1, 3, 16].

In the current study, respondents reported high levels of stress and working overtime. Many residents reported frequent consideration of quitting their jobs or changing careers, consistent with previous studies examining potential exit-strategy planning among surgical residents [17, 18]. As in the present study, Kiyasu et al. found that 33% of Japanese surgical residents were considering exit strategies. The reason for this is primarily due to workplace harassment [19]. Shaheen et al. report similar findings among surgical residents in Saudi Arabia, indicating a global trend in the surgical residency work environment [20]. The mentioned studies corroborate these assessments and confirm an existential problem in the work environment across various surgical fields at the global and national levels.

Although surgical professions are mainly associated with hands-on surgical tasks, the presented study shows different results. Many surgical residents reported that a significant portion of the daily workload consists of administrative and bureaucratic tasks. Interestingly, these tasks were rated as the most time-consuming aspect of the daily work in the current study, often exceeding the time spent on surgical training or direct patient care. This is not an isolated report, and similar findings are present in the current literature [9, 21]. Similarly, several large-scale surveys in the medical field reported that 70% of respondents stated they spend three hours or more each day on administrative and bureaucratic tasks [22]. These findings highlight an existing imbalance between surgical hands-on tasks and direct patient care and administrative obligations in the workflow of surgical residents.

However, despite high workloads, surgical residents reported strong interpersonal support within their teams in the current survey. Collegial solidarity was also evident in their professional relationships with senior surgeons. This is a key factor in creating a healthy work environment, as strong interpersonal support can result in higher work-related satisfaction [23]. The positive team dynamic indicates that workload-related stress is likely caused more by structural issues than by interpersonal conflicts [24]. Furthermore, positive experiences with mentoring and respectful interaction often stem from strong interpersonal relationships, which can be fostered by a supportive local environment and individual commitment [11].

Many respondents emphasized the need for more structured mentoring and the implementation of progressive, competency-based training pathways. Integrating targeted interventions such as mentoring and coaching may offer a promising approach to simultaneously strengthen training structures and improve leadership competencies in clinical settings [25]. The lack of feedback mechanisms limited operational involvement, and insufficient improvements to the training environment can contribute to growing dissatisfaction with surgical training. Eliminating these factors and structurally building on positive elements could offer a targeted opportunity to raise the quality to a new level [9, 26].

Most respondents perceived that there was no adequate compensation for personal burdens through structural measures, such as financial remuneration or paid time off. The implementation of such compensatory mechanisms might help mitigate stress and reduce the risk of early attrition [27]. Although no direct correlation can be established within the scope of this survey, the reported dissatisfaction levels and the high turnover rate expected for the coming years could indicate a relevant effect. This potential link must be investigated further, as it can only be inferred indirectly from the available results and is crucial for understanding fluctuation.

This aligns with prior research on surgical training programs, where many assistant doctors felt unprepared to assume clinical leadership roles, highlighting a lack of progressive, competence-based training pathways [28]. In addition, surgical training often takes longer than planned, with 75% of cases where residency must be extended due to insufficient surgical experience, which appropriate or increased training opportunities could help alleviate [28, 29]. There are promising trends in tackling the issue of inadequate structured training, such as the modified Delphi consensus on developing a national training curriculum for minimally invasive and robotic-assisted surgery by Huber et al. [30]. This seems to be an accelerator towards improving surgical education and training.

Respondents also voiced concerns about the healthcare system’s capacity to manage changing demographics, including an aging population and physician shortages, as well as its ability to adapt to workload fluctuations during crises. These concerns were partly confirmed during the COVID-19 pandemic, when heightened stress on the system increased dissatisfaction among key roles like surgeons and anesthesiologists [31, 32]. Furthermore, Germany has one of the lowest rates of medical graduates per inhabitant in the EU (12.4 per 100.000), with 31% of doctors aged 55 and above [22]. This supports the notion that the current healthcare system appears to fall short of its full potential, and high staff turnover in clinical surgical departments remains a significant issue.

There is an estimated surgical workforce shortage, along with an increased workload of 10–50% in surgical specialties [33]. These predictions highlight the importance of regularly evaluating working conditions and current opinions on work conditions among surgical residents to prevent workforce shortages in the surgical field.

The data collected highlight the challenges of surgical residency in university hospitals in Germany and align with previously published findings. Furthermore, initiatives for possible adapted implementation measures have already been described in the literature [29, 34–36]. The presented results can serve as guidelines for developing solutions that provide a basis for standardizing and transferring technical and interpersonal best practices, facilitating their context-specific implementation. Given that the issue is known and potential solutions have been suggested, the question arises of why earlier findings have not yet gained wider recognition or been implemented in clinical practice. To close this gap, investigations should be encouraged to identify possible reasons for the slow pace of adaptation or lack of acceptance, such as the need for implementation in different institutional and regional contexts.

### Limitations

The present study was designed as a cross-sectional, descriptive, hypothesis-generating survey, aiming to provide an initial overview of surgical training conditions among resident surgeons at German university hospitals. The sample size does not permit statistically powered subgroup analyses stratified by surgical specialty or institution type. Selection bias cannot be excluded, and the findings should therefore be interpreted as a preliminary, exploratory characterization of surgical training conditions rather than as representative conclusions for the entire German surgical workforce.

The statistical analysis did not account for the experiences of residents who responded partially or not at all. There are several possible reasons for partial or non-response. These groups may have had less time due to a high workload or been more socially oriented than others who participated in full-length sessions. Additionally, participation in the study might have been seen as unnecessary if the individuals thought their working conditions were acceptable or good. However, the consistent responses from survey participants across various hospitals and regions, as well as their alignment with previous studies, suggest a shared perspective among resident surgeons.

Furthermore, although the study was primarily designed to assess resident surgeons at university hospitals, 33 respondents from peripheral hospitals were retained in the final analysis. It is assumed that these respondents were forwarded the questionnaire by colleagues at the university hospitals in good faith to support the research. Since all peripheral respondents completed the survey in full, their responses were included to allow for a broader characterization of surgical training conditions beyond the university hospital setting.

In terms of generalizability, the study offers one additional limitation. It has focused on the surgical residents at the university hospital. Even though this has been done intentionally due to unique requirements, in terms of surgical, teaching, and academic endeavors that surgical residents at the university hospital encounter, it still limits the results on a specific population group. As a result, the present findings do not yet fully capture the breadth of perceptions of surgical working conditions among resident surgeons across all hospital types and do not permit subgroup analyses by career stage, institution, or surgical discipline. Future studies should aim to engage with all types of surgical centers and actively involve program directors as multipliers in their recruitment strategy, as their involvement has the potential to substantially increase participant numbers through targeted dissemination within their respective departments. This would allow a more comprehensive and representative picture of surgical training conditions to be obtained across the full spectrum of hospitals contributing to public healthcare in Germany. As a result, the present findings do not yet fully capture the breadth of perceptions regarding surgical working conditions among resident surgeons across all hospital types and do not permit subgroup analyses by career stage, institution, or surgical discipline.

In the future, conducting annual surveys would enable tracking changes in training conditions, workload, and career satisfaction over time, offering important insights into long-term trends in the German surgical workforce.

## Supplementary Information

Supplementary Material 1.

Supplementary Material 2.

Supplementary Material 3.

Supplementary Material 4.

Supplementary Material 5.

Supplementary Material 6.

## Data Availability

The raw participant data will not be openly published but can be obtained from the corresponding author upon reasonable request.
