# Supplementary material for: Workload demands, surgical education and training among resident surgeons in Germany: a cross-sectional questionnaire-based study
Source: BMC Med Educ. 2026 Oct 19;26:1682. doi: 10.1186/s12909-026-10519-w (PMC13681801; doi:10.1186/s12909-026-10519-w)
Supplement: Supplementary file 1 [file 12909_2026_10519_MOESM1_ESM.pdf]

Checklist for Reporting Results of Internet E-Surveys (CHERRIES)

Item Category	***Checklist Item***	***Explanation***
Design	Describe survey design	The study is a convenience sample. Researchers distributed questionnaire links to all universities (i.e., 63 universities) in Jilin Province. Each university informs the students to fill in and the subjects voluntarily filled out the questionnaire.
IRB (Institutional Review Board) approval and informed consent process	IRB approval	Jilin University granted ethical approval for this study.
	Informed consent	The subjects were informed of the purpose of the survey, anonymity, confidentiality, and voluntary principles before responding. In addition, the subjects were informed of the bonus amount and quota set by the questionnaire.
	Data protection	The questionnaire asked subjects to voluntarily fill in contact information such as emails to facilitate giving out bonuses. The personal information was only known to the person collecting the data. We only provided a database of deleted contacts for analysis by researchers.<br>The collected data was stored in the specific computer serve in the Vanke School of public health, Tsinghua University. Only specific personnel who have signed a confidentiality agreement can view and analyze the data.
Development and pretesting	Development and testing	Respondents' data were collected via Credamo (www.credamo.com). Before formal data collection, participants were invited to answer the questionnaire for testing, and the questionnaire was revised based on their feedback.
Recruitment process and description of the sample having access to the questionnaire	Open survey versus closed survey	Open survey. Students can answer the questionnaire by scanning the quick response (QR) code.
	Contact mode	Initial contact was made by filling in the questionnaire via the QR code on the promotional poster.
	Advertising the survey	We promoted the survey through the official account of online platforms (such as the WeChat official account)

Survey administration	Web/E-mail	The survey was published on an online questionnaire platform. Universities send questionnaire links to students through websites and social software chat groups (e.g. WeChat) for filling in.
	Context	The commercial platform specializes in publishing online questionnaires. We only used it for data collection.
	Mandatory/voluntary	The survey was voluntary.
	Incentives	The survey has set up cash rewards to encourage everyone to participate. The bonus settings are as follows:<br>Cash prizes included 1,250 slots for 20 Yuan (~ 3 USD), 50 slots for 200 Yuan (~ 31 USD) prize, one slot for 500 Yuan (~78 USD) prize, and one slot for 2000 Yuan (~314 USD).
	Time/Date	This data was collected between October 26th and November 18th, 2021.
	Randomization of items or questionnaires	Question order was not randomized.
	Adaptive questioning	There were adaptive questions in the questionnaire so that respondents can jump past unnecessary questions based on their answers. For example, if the subjects answered that they did not have any smoking behavior, they did not answer the smoking addiction scale.
	Number of Items	The most had 287 questions , and the least had 247 questions.
	Number of screens (pages)	4 screens.
	Completeness check	Technically, it is possible to do completeness checks before the questionnaire is submitted, and selecting a response option should be enforced, except for questions asking for contact information.
	Review step	Respondents were not allowed to go 'back' in the survey. However, they were asked to confirm that the current answer was correct before clicking to enter the next page.

Response rates	Unique site visitor	N/A.
	View rate (Ratio of unique sur- vey visitors/unique site	The survey is voluntary. The system cannot record the number of unique visitors, so the view rate

	visitors)	cannot be calculated.
	Participation rate (Ratio of unique visitors who agreed to participate/unique first survey page visitors)	The system cannot record the number of people to fill in and the number of visitors, so the participation rate cannot be calculated.
	Completion rate (Ratio of users who finished the survey/users who agreed to participate)	This was a voluntary questionnaire. By default, the participants submit questionnaires on behalf of their consent to participate, so the completion rate cannot be calculated.

Preventing multiple entries from the same individual	Cookies used	Cookies were not used.
	IP check	As the subjects are college students, they usually use the campus network, so the IP address may be repeated. So we didn't check the participant's IP.
	Log file analysis	N/A
	Registration	N/A

Analysis	Handling of incomplete questionnaires	Completed surveys were analyzed. For a small amount of missing data, the average value interpolation method is used to fill in.
	Questionnaires submitted with an atypical timestamp	The system has a cache mechanism to ensure that participants can still answer after exiting the questionnaire system accidentally. Therefore, we did not exclude the subjects according to the filling time. However, we have set a deadline for data collection, and the data filled in after the deadline will not be included in the analysis.
	Statistical correction	Avoid the deviation of results by using appropriate statistical methods. For example, the dependent variable of this study was non normal distribution, so binary logistic regression was used instead of linear regression. In addition, the bias-corrected percentile Bootstrap test was used to extract 1,000 repetitions.
