# Supplementary material for: Workload demands, surgical education and training among resident surgeons in Germany: a cross-sectional questionnaire-based study
Source: BMC Med Educ. 2026 Oct 19;26:1682. doi: 10.1186/s12909-026-10519-w (PMC13681801; doi:10.1186/s12909-026-10519-w)
Supplement: Supplementary file 2 [file 12909_2026_10519_MOESM2_ESM.pdf]

Work-Life Balance, Training, Workload, Burnout, and Career Progression Among Resident Surgeons in Different Specialties at University Hospitals in Germany: A Questionnaire-Based Study

Dear residents,

thank you for taking the time to participate in our study. Participation is voluntary and you can stop participating at any time without consequences.

Our goal is to understand your perception of the surgical specialty and the associated workload. Your opinions and suggestions are important to us. The questionnaire you complete will be anonymised and used for educational research. Your answers cannot be assigned to you personally. Our goal is to identify possible approaches and integrate them into university training and everyday clinical practice.

Thank you for your participation.

There are 30 questions in this survey.

Demographic data

What is your current age?

*

❶ Choose one of the following answers
Please choose **only one** of the following:

- ◯ <20 years
- ◯ 20-30 years
- ◯ 31-40 years
- ◯ 41-50 years
- ◯ >50 years

Gender

*

❶ Choose one of the following answers
Please choose **only one** of the following:

- ◯ Female
- ◯ Male
- ◯ Non-binary
- ◯ Prefer not to say
- ◯ Other

In which year did you graduate from medical school?

*

Choose one of the following answers
Please choose **only one** of the following:

- After 01.01.2024
- Between 01.01.2014 - 31.12.2024
- Between 01.01.2004 - 31.12.2014
- Between 01.01.1994 - 31.12.2004
- Before 31.12.1994

In which German federal state is the university hospital where you are currently working located?

*

Choose one of the following answers
Please choose **only one** of the following:

- Schleswig-Holstein
- Mecklenburg-Vorpommern
- Hamburg
- Bremen
- Berlin
- Niedersachsen
- Brandenburg
- Thüringen
- Hessen
- Sachsen
- Sachsen-Anhalt
- Nordrhein-Westfalen
- Rheinland-Pfalz
- Saarland
- Bayern
- Baden-Würtemberg
- None of these/ I am currently not working in Germany

What is your current Surgical residency status?
*

❶ Choose one of the following answers
Please choose **only one** of the following:

- ◯ First-year resident
- ◯ Second-year resident
- ◯ Third-year resident
- ◯ Fourth-year resident
- ◯ Fifth-year resident
- ◯ Sixth-year resident
- ◯ Not in residency yet
- ◯ Residency already completed

Do you work at a university hospital or a peripheral hospital? *

❶ Choose one of the following answers
Please choose **only one** of the following:

- ◯ University Hospital
- ◯ Peripheral Hospital

Surgical Speciality

In which surgical speciality are you currently working?

Please choose from the list (in alphabetical order).

*

❗ Choose one of the following answers
Please choose **only one** of the following:

- ◯ Cardiac Surgery
- ◯ Dermatologic Surgery
- ◯ ENT (Ear, Nose, and Throat)
- ◯ General Surgery
- ◯ Gynecologic Surgery
- ◯ Maxillofacial and Oral Surgery
- ◯ Neurosurgery
- ◯
- ◯ Orthopedic and Trauma Surgery
- ◯ Pediatric Surgery
- ◯ Plastic, Aesthetic and Reconstructive Surgery
- ◯ Thoracic surgery
- ◯ Urologic Surgery
- ◯ Vascular Surgery
- ◯ Visceral surgery
- ◯ Ophthalmology
- ◯ Other field

Workload

Overall, how many hours do you typically work per week?

*

❗ Choose one of the following answers
Please choose **only one** of the following:

- ◯ Less than 40 hours
- ◯ Between 40-45 hours
- ◯ Between 46-50 hours
- ◯ Between 51-55 hours
- ◯ More than 55 hours

What is the average duration of the operations in which you take part?

*

❗ Choose one of the following answers
Please choose **only one** of the following:

- ◯ Less than one hour
- ◯ 1-3 hours
- ◯ 3-5 hours
- ◯ 5-10 hours
- ◯ More than ten hours
- ◯ During my residency, I have never been assigned to the operating theatre.

Please rate the following using this five-point scale:

1. Never
2. Rarely
3. Sometimes
4. Often
5. Always

*

Please choose the appropriate response for each item:

	1	2	3	4	5
**How often do you feel exhausted when you go home from work?**	○	○	○	○	○
**How often does the topic of overwork come up around colleagues?**	○	○	○	○	○
**How often per week do you have to take over tasks for your colleagues?**	○	○	○	○	○
**Do you feel well supported by your colleagues?**	○	○	○	○	○

New technologies

Have the following new technologies influenced the workload in surgery?

1. Worsened
2. Somewhat worsened
3. Neutral
4. Somewhat improved
5. Improved

*

Please choose the appropriate response for each item:

	1	2	3	4	5
**Digitization of patient files**	○	○	○	○	○
**Specialized surgical procedures**	○	○	○	○	○
**High-end tools in surgery (e.g., DaVinci)**	○	○	○	○	○

If you can think of another new technology influencing your current work, please indicate it below.

Please make sure to describe only the new technology and to give it with a number in brackets. The above numbering also applies here: 1. Worsened, 2. Somewhat worsened, 3. Neutral, 4. Somewhat improved, 5. Improved)

Please be careful not to mention any names, positions or other personal data. Valid answers should not be longer than 5 words.

Please write your answer here:

Worktime allocation

On average, how much of your work time is currently allocated to each of the following tasks?

Please rank the following activities based on the amount of time (in hours) you dedicate to them, starting with the activity you spend the most time on and ending with the activity you spend the least time on or have no time for.

*

❗ All your answers must be different and you must rank in order.

❗ Please select from 1 to 7 answers.

Please number each box in order of preference from 1 to 7

Please choose at least 1 items.

Please choose no more than 100 items.

☐ Direct patient-related non-surgical tasks, e.g. preoperative patient counselling

☐ Surgical training

☐ Mentoring and professional development

☐ Medical emergencies

☐ Operative (OR) tasks, Surgical Procedures

☐ Research activities

☐ Administrative tasks, e.g. documentation and paperwork

Overtime

In the following you have the opportunity to describe an additional field in which field you spend overtime. Please be sure to describe only the field you spend the most overtime. Please ensure not to mention any names, positions or other personal data. Valid answers would be no longer than 5 words.

Please write your answer here:

Satisfaction with current allocation

How satisfied are you with the current allocation of your work time for each of the following tasks?

1. Not satisfied
2. Barely satisfied
3. Neutral
4. Somewhat satisfied
5. Satisfied

*

Please choose the appropriate response for each item:

	1	2	3	4	5
**Research activities**	○	○	○	○	○
**Surgical training**	○	○	○	○	○
**Medical emergencies**	○	○	○	○	○
**Operative (OR) tasks, Surgical Procedures**	○	○	○	○	○
**Direct patient-related non-surgical tasks, e.g. preoperative patient counselling**	○	○	○	○	○

Please rate the following question about compensation in terms of time and salary.

1. Strongly Disagree
2. Disagree
3. Neutral
4. Agree
5. Strongly Agree

*

Please choose the appropriate response for each item:

	1	2	3	4	5
**Do you feel adequately compensated with paid time off?**	○	○	○	○	○
**Do you feel adequately compensated for your work financially?**	○	○	○	○	○

Medical consultation due to workload

During your residency, did you seek medical advice and receive a diagnosis of a condition related to your workload (e.g., burnout) because your workload was too excessive?

*

Choose one of the following answers
Please choose **only one** of the following:

- Yes
- No
- Prefer not to say

Surgical training curricula

Are you provided with a structured surgical training program that defines your goals after each year of residency?

*

Choose one of the following answers
Please choose **only one** of the following:

- Yes
- No

Are you provided with designated time to train in the surgical training center?

*

Choose one of the following answers
Please choose **only one** of the following:

- Yes
- No
- There is no surgical training center or training possibility available

Are you guaranteed to perform the required number of certain surgeries yearly to acquire the skills needed for your next residency level?

*

Choose one of the following answers
Please choose **only one** of the following:

- Yes
- No

How supported do you feel by your colleagues and superiors in learning specific surgical tasks and surgical training?

1. ... do not feel supported at all
2. ... feel insufficiently supported
3. ... feel somewhat supported, but not at the desired level of my progress
4. ... feel supported enough to progress in my training
5. ... feel very supported

*

Please choose the appropriate response for each item:

	1	2	3	4	5
**I feel/do not feel ...**	○	○	○	○	○

How well do you feel prepared for the next level of your residency/being an attending surgeon?

1. ... do not feel prepared at all
2. ... do not feel prepared sufficiently
3. ... feel somewhat prepared
4. ... feel prepared enough
5. ... feel very well prepared

*

Please choose the appropriate response for each item:

	1	2	3	4	5
**I feel/do not feel ...**	○	○	○	○	○

Is there a structured curriculum for the surgical training center designed to improve your surgical skills?

*

❗ Choose one of the following answers

Please choose **only one** of the following:

- ◯ Yes
- ◯ No

If there is not a sufficient training structure, what would you like to implement in your current surgical training?

*

❗ Check all that apply

Please choose **all** that apply:

- ☐ Designated time during work hours for surgical training
- ☐ Structured training curriculum
- ☐ Designated surgical mentor
- ☐ Designated space and equipment
- ☐ There is sufficient training structure at my hospital
- ☐ Other (please specify in the following box)

If you chose "other (please specify in the following)" above please indicate it below. Please make sure to describe only and shortly what your are missing for sufficient training.

Please be careful not to mention any names, positions or other personal data. Valid answers should not be longer than 5 words.

Please write your answer here:

Research, mentoring and future aspects

Do you have designated time outside of your residency duties to perform research within your residency program?

*

Choose one of the following answers

Please choose **only one** of the following:

- Yes
- No

Are you provided with structured mentorship in your research?

*

❗ Choose one of the following answers

Please choose **only one** of the following:

◯ Yes

◯ No

Please answer the following questions using this five-point scale:

1. Strongly disagree
2. Disagree
3. Neutral
4. Agree
5. Strongly agree

*

Please choose the appropriate response for each item:

	1	2	3	4	5
**I feel well prepared to conduct independent research.**	○	○	○	○	○
**I am optimistic about the future prospects of my surgical career.**	○	○	○	○	○
**I am considering exit strategies after residency or within the next 3 years.**	○	○	○	○	○
**I believe efforts to attract and train the next generation of surgeons in Germany are effective and well-supported.**	○	○	○	○	○
**I feel confident about my ability to adapt to changes in the healthcare system.**	○	○	○	○	○
**I am optimistic about future work-life balance as my career progresses.**	○	○	○	○	○

Please answer the following questions using this five-point scale:

1. Strongly disagree
2. Disagree
3. Neutral
4. Agree
5. Strongly agree

*

Please choose the appropriate response for each item:

	1	2	3	4	5
**My surgical career has potential for professional growth and development.**	○	○	○	○	○
**I expect the workload for surgeons to increase significantly in the coming years.**	○	○	○	○	○
**I believe that telemedicine and remote monitoring advancements will significantly influence how patient care is delivered in surgery.**	○	○	○	○	○
**The healthcare system in Germany is well-prepared to adopt and incorporate innovations in surgical technologies.**	○	○	○	○	○
**I feel confident in my ability to adapt to and utilize new technologies in my surgical procedures.**	○	○	○	○	○

**I believe that there are enough staff to effectively manage the demands of the expected changes in patient care due to demographic changes.**	○	○	○	○	○
**I believe that there are enough staff to effectively manage the demands of the expected technological changes in patient care.**	○	○	○	○	○

Last question

Thank you for answering the previous questions in the questionnaire. Finally, we would like to ask for your consent. In the following field, you can choose whether you agree to use your anonymized data to process scientific questions and their publication.

I agree with the scientific use of my data. *

Choose one of the following answers

Please choose **only one** of the following:

- ○ Yes
- ○ No

Thank you for your participation.

Best wishes,

Amila Cizmic, Philipp Wise and Hans Christian Schmidt

Submit your survey.
Thank you for completing this survey.
